# Development and psychometric validation of a questionnaire assessing perception and acceptance of micronutrient-fortified bouillon cubes among non-index household members aged ≥ 15 years in northern Ghana

**DOI:** 10.1186/s12889-025-26144-z

**Published:** 2026-01-07

**Authors:** Felix Kwaku Kyereh, Agartha N. Ohemeng, Reina Engle-Stone, K. Ryan Wessells, Sika M. Kumordzie, Charles D. Arnold, Jennie N. Davis, Emily R. Becher, Ahmed D. Fuseini, Kania W. Nyaaba, Xiuping Tan, Stephen A. Vosti, Seth Adu-Afarwuah

**Affiliations:** 1 Department of Nutrition and Food Science, University of Ghana, Legon, Accra Ghana; 2 Department of Nutrition and Institute for Global Nutrition, University of California, Davis, CA USA; 3 Department of Agricultural and Resource Economics, University of California, Davis, CA USA

**Keywords:** Micronutrient deficiencies, Fortified bouillon cubes, Perception and acceptance, Questionnaire validation, Public health nutrition, Ghana

## Abstract

**Background:**

Micronutrient deficiencies remain a major public health issue in West Africa, contributing to anaemia, impaired cognitive development, and increased infection risk. Bouillon cubes, widely consumed in the region, offer a culturally appropriate means of micronutrient delivery. However, no validated instrument exists to assess the psychosocial and behavioural factors influencing household acceptance of fortified bouillon cubes. This study aimed to develop and validate a questionnaire to assess perceptions and acceptance of micronutrient-fortified bouillon cubes among non-index household members (NIHMs) in northern Ghana.

**Methods:**

A 29-item questionnaire was developed based on the Theory of Planned Behaviour and the Health Belief Model. Development involved a literature review, expert consultation, and pretesting with 18 adults. The instrument included 26 five-point Likert-scale items and 3 categorical items. One NIHM (i.e., household members aged ≥ 15 years who were not enrolled as primary trial participants) was randomly selected per household using the Kish method. The questionnaire was administered to 742 NIHMs within 1–2 months of intervention initiation in a double-blind randomised controlled trial comparing multiple micronutrient-fortified versus iodine-only bouillon cubes in Kumbungu and Tolon districts. Of these, 731 NIHMs were retained for analysis, with the dataset divided into exploratory (*n* = 292) and confirmatory (*n* = 439) factor analyses. Psychometric properties were evaluated using Cronbach’s alpha, composite reliability (CR), and average variance extracted (AVE).

**Results:**

Participants (mean age = 40.8 ± 17.4 years) were 58.8% female, 74.8% without formal education, and 38.4% were the primary household cooks. Exploratory factor analysis identified two constructs, Perception (8 items) and Acceptance (11 items), explaining 56% of variance. Confirmatory factor analysis retained 18 items (Perception = 8; Acceptance = 10) and showed good model fit (chi-square/df = 1.91, root mean square error of approximation [RMSEA] = 0.04, comparative fit index [CFI] = 0.98, Tucker–Lewis index [TLI] = 0.98, and standardized root mean square residual [SRMR] = 0.06). Internal consistency was acceptable (Cronbach’s alpha: Perception = 0.71; Acceptance = 0.72; CR = 0.86 and 0.82, respectively). AVE was 0.52 for Perception and 0.46 for Acceptance. Discriminant validity was supported.

**Conclusion:**

The validated 18-item questionnaire shows acceptable psychometric properties and offers a standardised tool for assessing household perception and acceptance of fortified foods, suitable for programme design, evaluation, and behavioural monitoring in bouillon-consuming settings.

**Supplementary Information:**

The online version contains supplementary material available at 10.1186/s12889-025-26144-z.

## Background

Micronutrient deficiencies (MNDs), commonly referred to as hidden hunger, remain a widespread public health challenge, particularly affecting women of reproductive age and preschool children in West Africa. These deficiencies contribute significantly to anaemia, impaired cognitive development, and increased vulnerability to infections [39, 56, 71]. In the northern regions of Ghana, dietary reliance on nutrient-poor staples, compounded by socioeconomic constraints, continues to sustain high rates of MNDs [48, 63, 67].

Large-scale food fortification is a proven strategy to mitigate MNDs and has showed population-level benefits in low- and middle-income countries [47, 50, 64]. However, in Ghana, the implementation of fortification initiatives has been constrained by regulatory challenges and limited access to adequately fortified products [46, 63]. The issue is not that households reject fortified versions, but rather that the food vehicles they routinely consume, such as oil and wheat flour, are often not adequately fortified. According to the Ghana Micronutrient Survey [63], only 56% of oil samples nationally, 36% in the Northern Belt, and less than 6% of wheat flour samples met fortification standards [65]. Bouillon cubes, consumed regularly by nearly all households across different socioeconomic groups, represent a culturally familiar and feasible means of micronutrient delivery. They offer the advantage of requiring minimal behavioural change in food preparation practices [1, 2, 21, 34, 43, 68].

Building on this premise, the Condiment Micronutrient Innovation Trial (CoMIT) conducted a community-based, double-blind, randomised controlled trial in the Kumbungu and Tolon districts of northern Ghana to assess the impact of multiple micronutrient-fortified bouillon cubes on nutritional status among women of reproductive age (15–49 y) and pre-school aged children (2–5 y) enrolled in the trial [22]. *Non-index household members* (NIHMs) were defined as individuals aged ≥ 15 years living in the households of the trial participants but not themselves enrolled in the trial. Typically, these include household heads who control staples and cash for food, and primary cooks, usually women, who decide on specific dishes and their preparation [15, 25, 53, 69, 74]. Their perceptions and acceptance of fortified bouillon cubes are key determinants of the reliability and effectiveness of fortification interventions at the household level. While NIHMs are key to sustaining fortified product use, no validated, theory-driven tool exists in sub-Saharan Africa to measure their perceptions and acceptance of the micronutrient-fortified bouillon cubes. Understanding these perceptions is important for designing interventions that are both acceptable and sustainable.

Therefore, this study aimed to develop and psychometrically validate a quantitative questionnaire to assess household perceptions and acceptance of micronutrient-fortified bouillon cubes. The validated tool is intended to support the evaluation and design of fortification interventions and inform public health strategies targeting MNDs in low-resource settings.

## Methods

### Study design and setting

This questionnaire validation was conducted as a sub-study within the Condiment Micronutrient Innovation Trial (CoMIT). Briefly, CoMIT was a double-blind, community-based randomised controlled trial conducted at 16 sites in the Kumbungu and Tolon districts of the Northern Region of Ghana (trial registration: ClinicalTrials.gov NCT05178407; Pan‑African Clinical Trial Registry PACTR202206868437931) and detailed in the published protocol [22] The trial assessed the impact of household use of bouillon cubes fortified with multiple micronutrients (iodine, vitamin A, folic acid, vitamin B12, iron, and zinc) compared to control cubes fortified with iodine alone. Outcomes included haemoglobin levels and selected biomarkers among three groups of index participants: non-pregnant, non-lactating women of reproductive age; lactating women 4–18 months postpartum; and children aged 2–5 years.

Households were randomised using a computer-generated block design, and blinding was maintained through identical packaging. Bouillon cubes were distributed biweekly, based on household size, over a nine-month period.

### Study participants and recruitment

Participants in this sub-study were non-index household members (NIHMs) aged 15 years or older who regularly shared meal in a day with the index participant, were not enrolled in the main trial, and provided written informed consent. For those aged 15–17 years, assent was obtained in addition to parental consent or that of a caregiver. Individuals who declined to provide consent or assent were excluded. Following household consent and recruitment of the index participant, one NIHM was randomly selected per household using the Kish method [45], based on the household roster compiled during the recruitment of the index participant. Selecting one NIHM per household helped avoid intra-household response dependence, while random selection minimised selection bias and ensured that perspectives were captured from a diverse range of household members beyond household heads or primary cooks.

### Theoretical framework

The questionnaire development was guided by two dual constructs; Theory of Planned Behaviour (TPB) and the Health Belief Model (HBM) [4, 52]. The TPB posits that behavioural intentions are shaped by attitudes**,** subjective norms**,** and perceived behavioural control. In this study, attitudes captured evaluations of sensory qualities and satisfaction with the micronutrient-fortified bouillon cubes, subjective norms reflected perceived social approval, and perceived behavioural control represented perceived ease of routine household use.

The HBM complements the TPB by emphasising beliefs about benefits**,** barriers**,** and self-efficacy in health-motivated behaviour. Perceived benefits were operationalised as willingness to continue using or purchasing the cubes, interpreted as a proximal indicator of value recognition, given that long-term health effects were not yet observable. Perceived barriers captured potential sensory or experiential obstacles, while cues to action and self-efficacy reflected household readiness and confidence to use the cubes routinely.

Integrating both frameworks allowed a comprehensive yet concise assessment of cognitive, social, and health-related determinants of fortified-cube acceptance and use.

### Questionnaire development and pretesting process

A literature review was conducted to identify key constructs from the TPB and the HBM relevant to the adoption of micronutrient-fortified foods. The review focused on fortified products such as condiments, seasonings, rice, and complementary foods, examining factors including flavour preferences, compatibility with traditional diets, cultural attitudes, and perceived health benefits [3, 20, 24, 66, 69]. Based on these domains, an initial pool of 33 items was generated. An interdisciplinary panel from the CoMIT research team, comprising experts in nutrition, public health, consumer behaviour, and statistics, evaluated the items for clarity and relevance. This process reduced the pool to 29 items.

The instrument was forward-translated from English into Dagbani by two bilingual translators familiar with nutrition and public-health terminology, and then back-translated into English by an independent translator who was blinded to the original version. Discrepancies between the two English versions were reviewed by the research team until conceptual and linguistic equivalence was achieved, consistent with recommended cross-cultural adaptation procedures [9].

Translational validity was further assessed by pretesting the questionnaire with 18 adult household members (cooks, household heads, and formal workers aged 20–70 years) from two rural communities located approximately 15–20 km from the nearest main study sites. These communities were part of the intervention sites. They were chosen because they shared similar cultural and dietary practices but were excluded from the main data collection to prevent contamination. Participants were purposively recruited to represent household heads, primary cooks, and other adult household members. During the pretesting, participants evaluated the clarity and cultural appropriateness of the questionnaire. Feedback from this process prompted iterative refinements, including rewording several items for brevity, adjusting response options, and clarifying the scale definitions before finalisation.

The pretest sample size (*n* = 18) was sufficient for assessing face and translational validity**,** ensuring clarity and cultural appropriateness of items prior to large-scale validation. While adequate for identifying issues of comprehension and wording, the small pretest sample may not have captured subgroup-specific interpretations and was therefore not used for psychometric validation. Construct validity was subsequently examined in the full study sample through exploratory and confirmatory factor analyses. The final questionnaire consisted of 26 items measured on a five-point Likert scale (1 = completely disagree to 5 = completely agree, with an additional non-applicable option), two categorical items, and one binary item. The orignal unvalidated instrument is provided as supplementary material [see Additional file 1]. 

### Data collection procedure

Trained fieldworkers, who were instructed in study-specific ethical procedures, including avoiding leading questions, ensuring cultural sensitivity, and maintaining participant confidentiality, administered the questionnaire through face-to-face interviews using tablet-based data entry (SurveyCTO). Background information on participants, including age, sex, household cooking role, education, and occupation, was also recorded. Supervisors reviewed datasets daily to ensure data quality and adherence to the study protocol. Data collection occurred between March and September 2023, and for each household, the questionnaire was administered within the first two months after the household began receiving the bouillon intervention. Because data were collected at this early stage, long-term health effects of fortification could not yet be observed; responses therefore reflect short-term perceptions and behavioural intentions rather than measurable health outcomes.

### Data processing and analysis

Data were collected from 742 participants; after excluding 11 incomplete or inconsistent responses, 731 valid records remained for analysis. Negatively framed items were reverse-scored, and three categorical items were recoded to align with the five-point Likert scale for inclusion in factor analyses.

Five items showing minimal response variation (over 95% identical responses) were removed because they lacked discriminative value [18, 33]. Outliers were assessed using univariate (Z-scores) and multivariate (Mahalanobis distance) criteria. To minimise their influence without losing statistical power, extreme values were adjusted through Winsorisation (± 3 SD), which retains all observations while reducing distortion from extreme responses [29, 41, 60].

The remaining 24 items were standardised (Z-scores, mean = 0, SD = 1) to ensure comparability across scales. Mardia’s test indicated deviations from multivariate normality, supporting the use of robust estimation methods [11, 40]. The final dataset (*n* = 731) was randomly split into two subsets: 40% (*n* = 292) for exploratory factor analysis (EFA) and 60% (*n* = 439) for confirmatory factor analysis (CFA). Randomisation was performed using a fixed seed to ensure reproducibility [35]. The EFA sample size exceeded the recommended criterion of 5–10 respondents per item [70], and the CFA sample exceeded the recommended minimum of 200–300 participants for stable model estimation [33, 55]. Background characteristics of participants were summarised using descriptive statistics. Analyses were conducted using R software (version 4.3.1) and the *lavaan* package in RStudio [38].

### Exploratory Factor Analysis (EFA)

Exploratory factor analysis was conducted to examine the underlying structure of the 24-item questionnaire and to explore whether the data supported a two-factor structure consistent with the theorised constructs of Perception and Acceptance [12]. Sampling adequacy was assessed using the Kaiser–Meyer–Olkin (KMO) measure, with values ≥ 0.80 considered meritorious for factor analysis [32]. Bartlett’s test of sphericity was also performed to assess whether the correlation matrix was suitable for factor analysis, with a significant result at *p* < 0.05 indicating that the inter-item correlations were sufficient for factor extraction [54, 72].

A two-factor solution was specified a priori based on theoretical expectations and further supported by parallel analysis, which indicated that only the first two eigenvalues exceeded the 95th-percentile random criterion. Principal Axis Factoring was used as the extraction method, and Direct Oblimin rotation was applied to allow for correlation between the two factors [5, 72]. Items were retained if their primary factor loading exceeded 0.40 and did not exhibit substantial cross-loading (≥ 0.30) on other factors, consistent with established guidelines for factor interpretability [28, 57, 59].

### Confirmatory factor analysis (CFA) and model validation

CFA was conducted to validate the two-construct model identified through EFA, assessing the relationships between observed items and their corresponding latent constructs (Perception and Acceptance) [11, 72]. Due to the ordinal nature of the data and significant deviations observed from multivariate normality, the Weighted Least Squares Mean and Variance-Adjusted (WLSMV) estimator was used [31, 37]. A scaling correction factor of 1.104 was applied to the chi-square statistic to ensure accurate interpretation of robust model fit indices [11, 36].

Model fit was evaluated using multiple standard indices. The Root Mean Square Error of Approximation (RMSEA) ≤ 0.06 indicated good fit, with values between 0.06 and 0.08 considered acceptable [27, 44]. Comparative Fit Index (CFI) and Tucker-Lewis Index (TLI) values ≥ 0.95 reflected excellent fit, while values between 0.90 and 0.95 were deemed acceptable. A Standardised Root Mean Square Residual (SRMR) ≤ 0.08 indicated adequate fit [26, 42]. The normed chi-square (χ2/df) was also reported, with values < 3 indicating acceptable model parsimony [7].

Beyond overall model fit, construct validity was evaluated to determine how well the observed items represented the intended theoretical constructs [10, 30]. Convergent validity, the extent to which items within a factor are correlated, was assessed using Average Variance Extracted (AVE), with values > 0.50 indicating adequate convergence [14]. Discriminant validity was assessed using the Fornell–Larcker criterion, whereby the square root of the AVE for each construct exceeded the inter-construct correlation [23]. Internal consistency and construct reliability were evaluated using Cronbach’s alpha and composite reliability (CR), with values ≥ 0.70 considered acceptable [28, 58]. To further assess convergent validity, factor loadings were examined to evaluate the strength of the relationships between observed items and their respective latent constructs. Standardised factor loadings were examined, with items loading ≥ 0.40 retained. Loadings ≥ 0.50 were considered acceptable, while values ≥ 0.70 indicated strong associations [28, 57].

Model refinement followed standard CFA procedures to improve overall fit. Correlation between the two constructs was permitted based on theoretical expectations. Items with standardised factor loadings below 0.40 or exhibiting substantial cross-loadings were considered for removal, provided that their exclusion did not compromise theoretical or content validity.

## Results

### Background characteristics

Table 1 presents the background characteristics of the study participants. A total of 731 participants were included (mean age = 40.8 ± 17.4 years), and 58.8% were female. Most were spouses (38.6%) or household heads (27.9%), and about four in ten (38.4%) served as the primary household cooks. Educational attainment was generally low, with three-quarters reporting no formal education. Nearly half (46.8%) were engaged in agriculture, and roughly one-third operated small businesses. The study population was largely homogeneous, with almost all participants identifying as Dagomba (98.9%) and Muslim (98.4%).

**Table 1:** Background characteristics of the study participants in the Northern region of Ghana^a^ (Total *n* = 731)

Variables/characteristics	Frequencies (%)
Age, *years (Mean* ± *S.D)*	40.8 ± 17.4
Sex	
Male	294 (41.2%)
Female	437 (58.8)
Relationship with household head (HH)	
Household head	204 (27.9)
Wife of HH	282 (38.6)
Father/mother of HH	69 (9.4)
Sons/daughters of HH	101 (13.8)
Siblings/mother in-law of HH	75 (10.3)
Position in household cooking	
Never cook	292 (40.0)
Primary cook	281 (38.4)
Occasional/rarely cook	158 (21.6)
Educational level completed	
No Formal Education	547 (74.8)
Basic Education	95 (13.0)
Senior High School or Higher	89 (12.2)
Ethnicity	
Dagomba	723(98.9)
Gonja/Mossi	8 (1.1)
Religion	
Islam	719 (98.4)
Christianity	12 (1.6)
Occupation	
Agriculture/Farming	342 (46.8)
Homemaker	122 (16.7)
Small Business Owner	211 (28.9)
Government/Private Employee	56 (7.7)

*Abbreviations*: *HH* Household Head

^a^Values are reported as means and standard deviation for continuous variable and count and percentages for categorical variables

### EFA

The KMO measure of sampling adequacy was 0.85, indicating that the sample was suitable for factor analysis. Bartlett’s test of sphericity was significant, χ2(276) = 2156.05, p < 0.001, confirming that the inter-item correlations were sufficient for factor extraction.

Exploratory factor analysis (EFA) revealed a two-factor structure, comprising Factor 1 (Perception) and Factor 2 (Acceptance), as shown in Table 2.

**Table 2:** Factor loadings and communalities from the exploratory factor analysis (*n* = 292)

Question (Item)	Item description	Factor 1 (Perception)	Factor 2 (Acceptance)	Communality
Q1	views unchanged since receiving study bouillon	0.70*	−0.04	0.498
Q2	Okay to use bouillon for all household members	−0.02	0.57*	0.331
Q3	Study bouillon smells same as regular bouillon	0.72*	−0.03	0.527
Q4	Study bouillon taste same as regular bouillon	0.74*	0.02	0.546
Q5	Study bouillon can be used daily	−0.04	0.50*	0.255
Q6	Study bouillon to be used only on some days	0.47*	−0.14	0.251
Q7	Study bouillon can be used multiple times daily	0.03	0.46*	0.213
Q10	Agree with those who think it's good	0.03	0.43*	0.184
Q11	Agree with those who think it's neutral	0.33	0.35	0.201
Q12	Agree with those who think it's bad	0.18	0.35	0.136
Q13	Cooking frequency changed since receiving bouillon	−0.09	0.16	0.037
Q14	Frequency of study bouillon use during cooking	0.01	0.02	0.000
Q16	Likes the smell of study bouillon	−0.12	0.44*	0.224
Q17	Likes the taste of study bouillon	−0.08	0.59*	0.371
Q18	Happy household receives study bouillon	0.00	0.55*	0.305
Q19	Study bouillon quantity received is sufficient	0.28	0.10	0.081
Q20	Enjoy foods prepared with study bouillon	0.06	0.67*	0.445
Q23	No personal problems observed with bouillon	0.61*	0.11	0.367
Q24	No problems observed by household members	0.61*	0.12	0.363
Q25	Positive views about study bouillon	0.09	0.57*	0.317
Q26	Does not want household to continue using bouillon	0.74*	−0.05	0.551
Q27	Thinks neighbours/friends would not like bouillon	0.76*	−0.05	0.584
Q28	Wants household to use bouillon in future	−0.08	0.59*	0.365
Q29	Would buy bouillon if sold in future	−0.05	0.66*	0.446

Factor loadings ≥ 0.40 are considered acceptable. Items marked with an asterisk (*) indicate those retained for their respective factors. Communality represents the proportion of variance in each item explained by the extracted factors

For the Perception factor, loadings ranged from 0.47 (Q6) to 0.76 (Q27). Eight items were retained: Q1, Q3, Q4, Q6, Q23, Q24, Q26, and Q27. For the Acceptance factor, loadings ranged from 0.43 (Q10) to 0.67 (Q20). Eleven items were retained: Q2, Q5, Q7, Q10, Q16, Q17, Q18, Q20, Q25, Q28, and Q29. No items cross-loaded at ≥ 0.30 on both factors.

Together, the two factors explained 56% of the total variance, with Acceptance accounting for 31% and Perception for 25%, which is within the acceptable range for behavioural constructs. Internal consistency was acceptable, with Cronbach’s alpha values of 0.71 for Perception and 0.72 for Acceptance.

### CFA

#### Final standardised factor loadings

Supplementary Table S1 presents the standardised factor loadings from the confirmatory factor analysis (CFA) model. All retained items exceeded the pre-established threshold of ≥ 0.40, supporting their inclusion in their respective latent constructs. Although 11 items initially loaded on the Acceptance construct in the EFA, one item (Q7) showed a low standardised factor loading (0.35) in the CFA and was excluded. This resulted in a final 10-item Acceptance construct, consistent with theoretical expectations.

For the *Perception* construct, standardised loadings ranged from 0.46 to 0.77, while for *Acceptance* they ranged from 0.48 to 0.69**.** These values indicate adequate item contributions and support the construct structure.

The final validated questionnaire measuring Perception and Acceptance is provided as supplementary material [see Additional file 2].

#### Model indices and validation results

Table 3 presents the model fit indices and construct validation results from the CFA. The CFA indicated acceptable to excellent model fit based on multiple criteria: χ2(134) = 255.94, *p* < 0.001; χ2/df = 1.91; RMSEA = 0.043 (90% CI: 0.035–0.052); CFI = 0.981; TLI = 0.977; and SRMR = 0.061. Although the chi-square test was statistically significant (*p* < 0.001), this result is expected with large samples and does not, by itself, indicate poor model fit. Other fit indices (CFI, TLI, RMSEA, SRMR) were within acceptable ranges, confirming that the overall model fit was adequate.

**Table 3:** CFA model fit indices and construct validity results

Index	Value	Acceptable Value
χ2 (df = 134)	255.94	Non-significant; sensitive to sample size
*p*-value	< 0.001	> 0.05 (not strict criterion due to sample size)
Chi-Square (χ2)/df	1.91	< 3.00
RMSEA (90% CI)	0.043 (0.035–0.052)	< 0.06 (good); < 0.08 (acceptable)
CFI	0.981	≥ 0.95 (good); ≥ 0.90 (acceptable)
TLI	0.977	≥ 0.95 (good); ≥ 0.90 (acceptable)
SRMR	0.061	< 0.08
CR – Perception	0.858	≥ 0.70
CR – Acceptance	0.824	≥ 0.70
AVE – Perception	0.520	≥ 0.50
AVE – Acceptance	0.460	≥ 0.50 *(marginal)*
Square root of AVE (Perception)	0.721	> Inter-construct correlation (0.45)
Square root of AVE (Acceptance)	0.678	> Inter-construct correlation (0.45)
Inter-construct correlation	0.450	< √AVE for each construct

*CR* Composite Reliability, *AVE* Average Variance Extracted, *RMSEA* Root Mean Square Error of Approximation, *CFI* Comparative Fit Index, *TLI* Tucker-Lewis Index, *SRMR* Standardised Root Mean Square Residual. CR > 0.70 and Square root of AVE > inter-construct correlation indicates good convergent and discriminant validity [23]

Composite reliability (CR) was 0.86 for the Perception construct and 0.82 for Acceptance**,** exceeding the recommended minimum of 0.70, thereby indicating strong internal consistency. The average variance extracted (AVE) was 0.52 for Perception and 0.46 for Acceptance. The Average Variance Extracted (AVE) for the Acceptance construct was 0.46, indicating marginal convergent validity but adequate composite reliability.

One item (Q7) from the Acceptance construct was removed due to a low standardised factor loading (0.35), which persisted despite allowing for correlated error terms. This removal improved model fit but did not compromise content validity, as the remaining items continued to represent all conceptual domains originally defined for the Acceptance construct. Its exclusion was justified by its weak association with the latent construct [14, 51].

Four items showed borderline factor loadings: Q5 (0.48), Q6 (0.48), Q10 (0.49), and Q24 (0.46). These items were retained because of their theoretical importance in capturing perceived behavioural control, normative approval, and experiential feedback, core constructs within the underlying behavioural frameworks (Theory of Planned Behaviour and Health Belief Model). Retaining them ensured that the multidimensional nature of the Acceptance construct was preserved without compromising overall model fit or conceptual validity.

The correlation between Perception and Acceptance was *r* = 0.45, indicating a moderate association. This suggests related but distinct constructs, consistent with the conceptual separation of beliefs and behavioural intentions proposed by the Theory of Planned Behaviour. However, discriminant validity was supported. Thus, the square root of the AVE was 0.72 for Perception and 0.68 for Acceptance, both exceeding the inter-construct correlation of 0.45, consistent with the Fornell–Larcker criterion. These results collectively support a two-factor model with acceptable reliability and validity, consistent with the theoretical expectations.

## Discussion

This study developed and validated an 18-item questionnaire to assess ‘Perceptions’ (8 items) and ‘Acceptance’ (10 items) of micronutrient-fortified bouillon cubes among non-index household members in northern Ghana. Factor analyses confirmed a two-factor structure explaining 56% of the variance, indicating a well-fitting and theoretically coherent model. Internal consistency was acceptable (Cronbach’s alpha: 0.71 for Perception, 0.72 for Acceptance; composite reliability: 0.86 and 0.82, respectively). Convergent validity was confirmed for Perception (AVE = 0.52) and was marginal for Acceptance (AVE = 0.46), while discriminant validity was supported by the Fornell–Larcker criterion.

A key strength was that the questionnaire was theory-driven, drawing on constructs from the Theory of Planned Behaviour and the Health Belief Model [4, 52]. The questionnaire was reviewed by experts, pretested, and field-tested within a randomised controlled trial context [10]. Both exploratory and confirmatory factor analyses were applied to independent samples. Robust estimation methods (WLSMV) were used to account for the ordinal nature of the data and non-normality, enhancing the validity of the findings [11, 36].

Although items Q5, Q6, Q10, and Q24 had comparatively lower factor loadings, they were retained due to their theoretical relevance in capturing perceived behavioural control, normative approval, and experiential feedback. The slightly sub-threshold AVE for the Acceptance construct (0.46) is acknowledged as a limitation, indicating moderate convergent validity but acceptable construct reliability. Despite this marginal value, the construct was retained because of its theoretical relevance and overall psychometric adequacy. The relatively low AVE likely reflects that some items captured overlapping influences, such as social norms and sensory appeal. This overlap may have reduced the proportion of variance explained by a single latent construct, despite adequate composite reliability. The removal of one item (Q7) during CFA did not compromise content validity, as the remaining items continued to represent all theoretical domains of the Acceptance construct. Although these indices lie at the lower bound of acceptability, they reflect common challenges in early-stage instrument validation and should be addressed through future item refinement. All factor loadings exceeded 0.40 and composite reliability was high (0.82), indicating acceptable internal consistency. Prior psychometric studies emphasise that composite reliability is often a more robust indicator than AVE when factor loadings are strong [14, 28]. This approach aligns with earlier food-related validations, where constructs with AVE < 0.50 were retained if supported by theory and strong reliability indices [13]. In this study, the Acceptance construct captured willingness, sensory appeal, and household-level behavioural intentions, dimensions central to sustained adoption of fortified foods. Excluding it would have compromised both conceptual completeness and programmatic utility.

The study sample showed limited variation in ethnicity, religion, and socioeconomic status, with most participants being Dagomba and Muslim. This homogeneity may have reduced cultural diversity in responses. Consequently, the findings may not generalise to more culturally diverse or urban populations, underscoring the need for replication in varied settings. Because of its cross-sectional design, this study can identify associations but not causal relationships or temporal stability [6]. However, the primary aim was to develop and validate a psychometric instrument rather than to infer causal directions. The small pretest sample may not have captured subgroup-specific interpretations, which could influence item comprehension. Test–retest reliability was not assessed, a major limitation since temporal stability remains untested; future studies should evaluate test–retest reliability to establish score consistency over time. Criterion validity was not assessed in this study, as no direct behavioural indicators (e.g., cube disappearance or household inventory) were available. The absence of criterion validation against observed household behaviours limits the ability to link reported perceptions to actual practice. Further work is underway to explore how perception and acceptance scores relate to nutritional outcomes such as haemoglobin and iodine status. Most respondents, approximately two-thirds (67%), were household heads or their spouses and were therefore actively involved in household food decisions, although a minority were not primary decision-makers. The random selection approach reduced selection bias and ensured representativeness. However, it may also have introduced variability in responses linked to differing levels of decision-making authority, which could attenuate associations with reported perceptions. Moreover, tool was administered early in the intervention (within two months), when direct health benefits were not yet observable. Administering the tool at this stage limited the ability to link perceptions and acceptance with objective nutritional outcomes. Future follow-up assessments could help determine whether early attitudes predict subsequent biochemical improvements.

The two-factor structure “Perception and Acceptance” reflects distinct but related behavioural dimensions, consistent with the TPB, which distinguishes between beliefs and behavioural intentions [4]. The moderate correlation (*r* = 0.45) supports this differentiation and aligns with findings from similar behavioural instrument validations [10, 19]. All factor loadings exceeded 0.40 and composite reliability was high (0.82), indicating acceptable internal consistency. Cronbach’s alpha values (0.71 and 0.72) were at the lower bound of the acceptable range but are typical for behavioural constructs of this nature. The total variance explained (56%) is considered adequate for instruments measuring psychosocial constructs, acknowledging that the remaining unexplained variance may reflect unmeasured contextual or individual influences. The study’s reliability values are within the acceptable range for new psychosocial scales, but they remain modest and should be improved through item refinement and retesting in diverse samples. Our findings are broadly comparable with other validated food-related instruments. For the East African Food Literacy Scale achieved Cronbach’s alpha of 0.71–0.78 with confirmed construct validity across Uganda and Kenya [73]. More recently, the Ultra-Processed Food Acceptance Scale in Spain and Portugal identified multiple acceptance dimensions [13].

Validated tools for assessing household perceptions and acceptance of fortified condiments are scarce in sub-Saharan Africa [16]. This instrument provides a reliable, context-specific measure that can support programme monitoring and design, especially in regions where bouillon cubes are widely used [21, 34]. Its use in a trial setting enhances its relevance for evaluating behavioural responses to fortified food interventions. Given that nearly 60% of respondents were female and many held primary cooking roles, gendered responsibilities may have influenced item interpretation and response patterns. These contextual factors are relevant for understanding how the questionnaire performs in similar household settings and should be examined in future validation studies across gender subgroups.

The validated questionnaire can be applied in both research and public health programme contexts to assess household acceptance and perception of fortified condiments. These constructs are relevant for informing communication strategies and intervention design [8]. In programme settings, the tool can support the planning and adaptation of fortification initiatives. It is relevant for ministries of health, non-governmental organization and other implementing partners. It may be used to identify specific concerns through item-level responses or to generate composite scores for monitoring and evaluation purposes. For example, low scores could flag households needing targeted engagement to address misconceptions about fortification interventions. Programmes may set pragmatic cut-offs (e.g., the lowest quintile) for targeted counselling. However, formal threshold validation is recommended before prespecifying programmatic triggers. The moderate association observed between perception and acceptance indicates that improving perceptions alone may not be sufficient to ensure adoption. Interventions should also address practical barriers, including taste preferences, cost, and cooking practices, to support sustained use [62]. Because fortified condiments are used at the household level, the views of all household members should be considered. This includes not only the primary beneficiaries but also others involved in food preparation and decision-making. Although the tool was developed for fortified bouillon cubes, the approach may be adapted for other fortified foods that are consumed at the household level. This flexibility makes it relevant for a range of food fortification programmes beyond bouillon.

Future research should assess this tool’s generalisability in different regions and cultural settings. Longitudinal studies can help evaluate changes over time in response to behaviour change interventions or market factors [49, 61]. Further validation is recommended, including predictive and criterion-related validity. These analyses could explore associations with both consumption behaviours and health outcomes [17, 33]. Measurement invariance across subgroups (e.g., gender, age, or district) was not tested in this initial validation. This limits the ability to confirm whether the constructs operate equivalently across groups. Future studies should assess factorial invariance to ensure the instrument’s comparability across diverse populations. Item refinement, particularly within the Acceptance construct, may also enhance measurement precision.

In summary, this 18-item instrument showed acceptable psychometric performance in a relatively homogeneous Ghanaian sample. While it offers a promising tool for assessing household perceptions and acceptance of fortified bouillon cubes, its use beyond similar contexts should be undertaken cautiously until further validation, including test–retest reliability, criterion validity, and measurement invariance analyses, is completed.

## Supplementary Information

Supplementary Material 1.

Supplementary Material 2.

Supplementary Material 3.

## Data Availability

The datasets generated and analysed during the current study are available from the corresponding author on reasonable request. The preliminary version of the questionnaire used during development is provided as supplementary material (Additional file 1). The final validated version of the questionnaire is provided as Additional file 2. Supplementary Table S1, which presents the standardised factor loadings for items retained in the final confirmatory factor analysis model, is also included as part of the supplementary materials.

## Abbreviations

AVE: Average Variance Extracted
CFA: Confirmatory Factor Analysis
CFI: Comparative Fit Index
CoMIT: Condiment Micronutrient Innovation Trial
CR: Composite Reliability
EFA: Exploratory Factor Analysis
HBM: Health Belief Model
MNDs: Micronutrient Deficiencies
NIHMs: Non-Index Household Members
RMSEA: Root Mean Square Error of Approximation
SRMR: Standardised Root Mean Square Residual
TLI: Tucker–Lewis Index
TPB: Theory of Planned Behaviour
WLSMV: Weighted Least Squares Mean and Variance-Adjusted
