# Supplementary material for: Development and psychometric validation of a questionnaire assessing perception and acceptance of micronutrient-fortified bouillon cubes among non-index household members aged ≥ 15 years in northern Ghana
Source: BMC Public Health. 2026 Jan 7;26:456. doi: 10.1186/s12889-025-26144-z (PMC12870002; doi:10.1186/s12889-025-26144-z)
Supplement: Supplementary file 1 — Supplementary Material 1. [file 12889_2025_26144_MOESM1_ESM.docx]

**Additional file 1:** **The questionnaires developed**

**Field worker will say:**

*I would like to know what you think and feel (or believe) about the CoMIT Project bouillon cubes (i.e. the bouillon your household is receiving from the CoMIT Project). I am going to read some questions or statements to you, and I would like you to tell me how you agree with those questions or statements. Most questions or statements have numbers 1 to 5, with* ***1 = Completely disagree; 2 = Disagree; 3 = Neither agree nor disagree; 4 = Agree; 5 = Completely agree.***

| # | **Question** | **Level of agreement*** | | | | | |
| --- | --- | --- | --- | --- | --- | --- | --- |
|  |  | **1** | **2** | **3** | **4** | **5** | **99** |
| Q1 | **What you used to think, feel, and do about bouillon cube use in household cooking has not changed since your household started receiving bouillon cubes from the study (CoMIT Project).**  *Enter the number corresponding to the participant’s level of agreement.* | \|__\| | \|__\| | \|__\| | \|__\| | \|__\| | \|__\|__\| |
| Q2 | **That it is okay to use the *study bouillon* *cubes* in cooking food meant for everyone in your household.**  *Enter the number corresponding to the participant’s level of agreement.* | \|__\| | \|__\| | \|__\| | \|__\| | \|__\| | \|__\|__\| |
| Q3 | **That the smell from the study bouillon cubes is not different from the other bouillon your household has used before.**  *Enter the number corresponding to the participant’s level of agreement.* | \|__\| | \|__\| | \|__\| | \|__\| | \|__\| | \|__\|__\| |
| Q4 | **That the taste of the study bouillon cubes is not different from the other bouillon your household has used before.**  *Enter the number corresponding to the participant’s level of agreement.* | \|__\| | \|__\| | \|__\| | \|__\| | \|__\| | \|__\|__\| |
| Q5 | **That the *study bouillon cubes* can be used in your household every day during the week.**  *Enter the number corresponding to the participant’s level of agreement.* | \|__\| | \|__\| | \|__\| | \|__\| | \|__\| | \|__\|__\| |
| Q6 | **That the *study bouillon cubes* should be used in your household in some, but not all, of the days of the week.**  *Enter the number corresponding to the participant’s level of agreement.* | \|__\| | \|__\| | \|__\| | \|__\| | \|__\| | \|__\|__\| |
| Q7 | **That the *study bouillon cubes* can be used in your household any number of times in a day.**  *Enter the number corresponding to the participant’s level of agreement.* | \|__\| | \|__\| | \|__\| | \|__\| | \|__\| | \|__\|__\| |
| Q8 | **That the *study bouillon cubes* can be used in any food being prepared in your household, if the person preparing the food chooses to do so.**  *Enter the number corresponding to the participant’s level of agreement.* | \|__\| | \|__\| | \|__\| | \|__\| | \|__\| | \|__\|__\| |
| *Read question 9 and enter the corresponding number of the participant’s response (1 = good, 2 = bad, 3 = neither good nor bad).* | | | | | | | |
| Q9 | Do you think that the study bouillon cubes are (1) *good* or (2) *bad* or (3) *neither good nor bad or* (99) not applicable? | \|__\| | \|__\| | \|__\| |  | | \|__\|__\| |
| *Say questions 10, 11, and 12 in reference to the 5 levels of agreements described above. Enter the number corresponding to the participant’s level of agreement.* | | | | | | | |
| \| **#Question** \| **1** \| **2** \| **3** \| **4** \| **5** \| **99** \| \| --- \| --- \| --- \| --- \| --- \| --- \| --- \| | | | | | | | |
| Q10 | How do you agree with those who think that the *study bouillon cubes* are good? | \|__\| | \|__\| | \|__\| | \|__\| | \|__\| | \|__\|__\| |
| Q11 | How do you agree with those who think that the *study bouillon cubes* are neither good nor bad? | \|__\| | \|__\| | \|__\| | \|__\| | \|__\| | \|__\|__\| |
| Q12 | How do you agree with those who think that the *study bouillon cubes* are bad? | \|__\| | \|__\| | \|__\| | \|__\| | \|__\| | \|__\|__\| |
| *Read question 13 and enter the number (1 or 2 or 3) corresponding to the participant's opinion.* | | | | | | | |
| Q13 | Do you think your household members (1) do more cooking since you started getting the study bouillon cubes than before, (2) cooking has not changed, or (3) you don’t know? | \|__\| | \|__\| |  | | | \|__\|__\| |
| *Read question 14 and stick to the responses provided in the question. Enter the number corresponding to the participant’s answer that he or she will select.* | | | | | | | |
| Q14 | Do you think your household uses the study bouillon (1) every time you/they cook, or (2) nearly every time you/they cook, or (3) some of the time you/they cook, (4) once in a while, or (5) you don’t know? | \|__\| | \|__\| | \|__\| | \|__\| | \|__\| | \|__\|__\| |
| *Read question 15 and enter the corresponding number of the participant’s response (1 = like, 2 = not like, 3 = don’t know).* | | | | | | | |
| Q15 | Do you (1) like or (2) not like the way bouillon is used in your household, or (3) you don’t know? | \|__\| | \|__\| |  | | | \|__\|__\| |

Acceptance towards study bouillon cubes

In this section, ask participants about their acceptance of study bouillon cubes. Say each question and refer to the level of agreement for the participant to select from. Next, fill in the box with the number that corresponds to the participant's level of agreement. Enter 99 “not applicable” if the participant is not willing to provide an answer. Do not leave any question unanswered.

**Field worker will say:**

*I would like to know what you say about your household's use of (or willingness to use) the CoMIT Project bouillon cubes (i.e., the bouillon your household is receiving from the CoMIT Project) now and in the future (acceptance). I am going to read you some statements. I would like you to tell me to what extent you agree with these statements. All the statements have numbers 1 to 5 representing your level of agreement, as follows:* ****Level of agreement: 1 = Completely disagree; 2 = Disagree; 3 = Neither agree nor disagree; 4 = Agree; 5 = Completely agree.***

| **#** | **Question** | **Level of agreement*** | | | | | |
| --- | --- | --- | --- | --- | --- | --- | --- |
|  |  | **1** | **2** | **3** | **4** | **5** | **99** |
| Q16 | You like the smell of the study bouillon cubes. | \|__\| | \|__\| | \|__\| | \|__\| | \|__\| | \|__\|__\| |
| Q17 | You like the taste of the study bouillon cubes. | \|__\| | \|__\| | \|__\| | \|__\| | \|__\| | \|__\|__\| |
| Q18 | You are okay or happy that your household receives the study bouillon | \|__\| | \|__\| | \|__\| | \|__\| | \|__\| | \|__\|__\| |
| Q19 | That the quantity of the study bouillon cubes your households receives is just fine. | \|__\| | \|__\| | \|__\| | \|__\| | \|__\| | \|__\|__\| |
| Q20 | That you and your household members like or enjoy foods prepared using the study bouillon cubes. | \|__\| | \|__\| | \|__\| | \|__\| | \|__\| | \|__\|__\| |
| Q21 | That you would like your household to also purchase non-study bouillon from the market. | \|__\| | \|__\| | \|__\| | \|__\| | \|__\| | \|__\|__\| |
| Q22 | That in the past month, you think your household should have purchased non-study bouillon from market. | \|__\| | \|__\| | \|__\| | \|__\| | \|__\| | \|__\|__\| |
| Q23 | That you have not observed any problems with using the study bouillon cubes in your household. | \|__\| | \|__\| | \|__\| | \|__\| | \|__\| | \|__\|__\| |
| Q24 | That your household members have not observed any problems with using the study bouillon cubes. | \|__\| | \|__\| | \|__\| | \|__\| | \|__\| | \|__\|__\| |
| Q25 | That you have good things to say about the study of bouillon cubes. | \|__\| | \|__\| | \|__\| | \|__\| | \|__\| | \|__\|__\| |
| Q26 | That you do not want your household to continue using the study bouillon. | \|__\| | \|__\| | \|__\| | \|__\| | \|__\| | \|__\|__\| |
| Q27 | That you do not think your neighbours or friends would like the study bouillon. | \|__\| | \|__\| | \|__\| | \|__\| | \|__\| | \|__\|__\| |
| Q28 | That you want your household to use the study bouillon in future if it is available. | \|__\| | \|__\| | \|__\| | \|__\| | \|__\| | \|__\|__\| |
| Q29 | That you would be interested in buying the study bouillon if it is sold in future. | \|__\| | \|__\| | \|__\| | \|__\| | \|__\| | \|__\|__\| |
