# Supplementary material for: Development and psychometric validation of a questionnaire assessing perception and acceptance of micronutrient-fortified bouillon cubes among non-index household members aged ≥ 15 years in northern Ghana
Source: BMC Public Health. 2026 Jan 7;26:456. doi: 10.1186/s12889-025-26144-z (PMC12870002; doi:10.1186/s12889-025-26144-z)
Supplement: Supplementary file 2 — Supplementary Material 2. [file 12889_2025_26144_MOESM2_ESM.docx]

**Additional file 2: Validated Perception Questionnaire**

In this section, ask participants about their perception of study bouillon cubes. Say each question and refer to the level of agreement for the participant to select from. Next, fill in the box with the number that corresponds to the participant's level of agreement. Enter 99 “not applicable” if the participant is not willing to provide an answer. Do not leave any question unanswered.

**Field worker will say:**

*I would like to know what you think and feel (or believe) about the CoMIT Project bouillon cubes (i.e. the bouillon your household is receiving from the CoMIT Project). I am going to read you some statements. I would like you to tell me to what extent you agree with these statements. All the statements have numbers 1 to 5 representing your level of agreement, as follows:* ****Level of agreement: 1 = Completely disagree; 2 = Disagree; 3 = Neither agree nor disagree; 4 = Agree; 5 = Completely agree.***

| **#** | **Question** | **Level of agreement*** | | | | | |
| --- | --- | --- | --- | --- | --- | --- | --- |
|  |  | **1** | **2** | **3** | **4** | **5** | **99** |
| Q1 | **What you used to think, feel, and do about bouillon cube use in household cooking has not changed since your household started receiving bouillon cubes from the study (CoMIT Project).**  *Enter the number corresponding to the participant’s level of agreement.* | \|__\| | \|__\| | \|__\| | \|__\| | \|__\| | \|__\|__\| |
| Q3 | **That the smell from the study bouillon cubes is not different from the other bouillon your household has used before.**  *Enter the number corresponding to the participant’s level of agreement.* | \|__\| | \|__\| | \|__\| | \|__\| | \|__\| | \|__\|__\| |
| Q4 | **That the taste of the study bouillon cubes is not different from the other bouillon your household has used before.**  *Enter the number corresponding to the participant’s level of agreement.* | \|__\| | \|__\| | \|__\| | \|__\| | \|__\| | \|__\|__\| |
| Q6 | **That study *bouillon cubes* should be used in your household in some, but not all, of the days of the week.**  *Enter the number corresponding to the participant’s level of agreement.* | \|__\| | \|__\| | \|__\| | \|__\| | \|__\| | \|__\|__\| |
| Q23 | **That you have not observed any problems with using the study bouillon cubes in your household.**  *Enter the number corresponding to the participant’s level of agreement.* | \|__\| | \|__\| | \|__\| | \|__\| | \|__\| | \|__\|__\| |
| Q24 | **That your household members have not observed any problems with using the study bouillon cubes.**  *Enter the number corresponding to the participant’s level of agreement.* | \|__\| | \|__\| | \|__\| | \|__\| | \|__\| | \|__\|__\| |
| Q26 | **That you do not want your household to continue using the study bouillon.**  *Enter the number corresponding to the participant’s level of agreement.* | \|__\| | \|__\| | \|__\| | \|__\| | \|__\| | \|__\|__\| |
| Q27 | **That you do not think your neighbours or friends would like the study bouillon.**  *Enter the number corresponding to the participant’s level of agreement.* | \|__\| | \|__\| | \|__\| | \|__\| | \|__\| | \|__\|__\| |

**Validated Acceptance Questionnaire**

In this section, ask participants about their acceptance of study bouillon cubes. Say each question and refer to the level of agreement for the participant to select from. Next, fill in the box with the number that corresponds to the participant's level of agreement. Enter 99 “not applicable” if the participant is not willing to provide an answer. Do not leave any question unanswered.

**Field worker will say:**

*I would like to know what you say about your household's use of (or willingness to use) the CoMIT Project bouillon cubes (i.e., the bouillon your household is receiving from the CoMIT Project) now and in the future (acceptance). I am going to read you some statements. I would like you to tell me to what extent you agree with these statements. All the statements have numbers 1 to 5 representing your level of agreement, as follows:* ****Level of agreement: 1 = Completely disagree; 2 = Disagree; 3 = Neither agree nor disagree; 4 = Agree; 5 = Completely agree.***

| **#** | **Question** | **Level of agreement*** | | | | | |
| --- | --- | --- | --- | --- | --- | --- | --- |
|  |  | **1** | **2** | **3** | **4** | **5** | **99** |
| Q2 | **That it is okay to use the *study bouillon* *cubes* in cooking food meant for everyone in your household.**  *Enter the number corresponding to the participant’s level of agreement.* | \|__\| | \|__\| | \|__\| | \|__\| | \|__\| | \|__\|__\| |
| Q5 | **That study *bouillon cubes* can be used in your household every day during the week.**  *Enter the number corresponding to the participant’s level of agreement.* | \|__\| | \|__\| | \|__\| | \|__\| | \|__\| | \|__\|__\| |
| Q10 | **How do you agree with those who think that study *bouillon cubes* are good?**  *Enter the number corresponding to the participant’s level of agreement.* | \|__\| | \|__\| | \|__\| | \|__\| | \|__\| | \|__\|__\| |
| Q16 | **You like the smell of the study bouillon cubes.**  *Enter the number corresponding to the participant’s level of agreement.* | \|__\| | \|__\| | \|__\| | \|__\| | \|__\| | \|__\|__\| |
| Q17 | **You like the taste of the study bouillon cubes.**  *Enter the number corresponding to the participant’s level of agreement.* | \|__\| | \|__\| | \|__\| | \|__\| | \|__\| | \|__\|__\| |
| Q18 | **You are okay or happy that your household receives the study bouillon.**  *Enter the number corresponding to the participant’s level of agreement.* | \|__\| | \|__\| | \|__\| | \|__\| | \|__\| | \|__\|__\| |
| Q20 | **That you and your household members like or enjoy foods prepared using the study bouillon cubes.**  *Enter the number corresponding to the participant’s level of agreement.* | \|__\| | \|__\| | \|__\| | \|__\| | \|__\| | \|__\|__\| |
| Q25 | **That you have good things to say about the study of bouillon cubes.**  *Enter the number corresponding to the participant’s level of agreement.* | \|__\| | \|__\| | \|__\| | \|__\| | \|__\| | \|__\|__\| |
| Q28 | **That you want your household to use the study bouillon in future if it is available.**  *Enter the number corresponding to the participant’s level of agreement.* | \|__\| | \|__\| | \|__\| | \|__\| | \|__\| | \|__\|__\| |
| Q29 | **That you would be interested in buying the study bouillon if it is sold in future**.  *Enter the number corresponding to the participant’s level of agreement.* | \|__\| | \|__\| | \|__\| | \|__\| | \|__\| | \|__\|__\| |
