# Supplementary material for: Development and psychometric validation of a questionnaire assessing perception and acceptance of micronutrient-fortified bouillon cubes among non-index household members aged ≥ 15 years in northern Ghana
Source: BMC Public Health. 2026 Jan 7;26:456. doi: 10.1186/s12889-025-26144-z (PMC12870002; doi:10.1186/s12889-025-26144-z)
Supplement: Supplementary file 3 — Supplementary Material 3. [file 12889_2025_26144_MOESM3_ESM.docx]

This table presents the standardised factor loadings for all items retained in the final two-factor CFA model assessing Perception and Acceptance of micronutrient-fortified bouillon cubes among non-index household members in northern Ghana. The analysis was conducted using the Weighted Least Squares Mean and Variance-Adjusted (WLSMV) estimator. Items with loadings ≥ 0.40 were retained as indicators of their respective latent constructs. One item (Q7: Study bouillon can be used multiple times daily) was excluded due to a low standardised loading (0.35) and theoretical redundancy. All retained items met the minimum loading threshold, supporting the two-construct measurement structure.

| **Supplementary Table S1. Standardised factor loadings for items retained in the final confirmatory factor analysis (CFA) model (n = 439)** | | | |
| --- | --- | --- | --- |
| **Perception construct** | **Loadings** | **Acceptance construct** | **Loadings** |
| Q1- Views unchanged since receiving study bouillon | 0.770 | Q2 – Okay to use bouillon for everyone | 0.525 |
| Q3 – Study bouillon smells same as regular bouillon | 0.756 | Q5 – Study Bouillon can be used daily | 0.476 |
| Q4 – Study bouillon taste same as regular bouillon | 0.719 | Q10 – Agree with those who think it's good | 0.486 |
| Q6 – Study bouillon to be used only on some days | 0.483 | Q16 – Likes the smell of study bouillon | 0.538 |
| Q23 – No personal problems observed with bouillon | 0.520 | Q17 – Likes the taste of study bouillon | 0.606 |
| Q24 – No problems observed by household members | 0.464 | Q18 – Happy household receives study bouillon | 0.578 |
| Q26 – Does not want household to continue using bouillon | 0.743 | Q20 – Enjoy foods prepared with study bouillon | 0.607 |
| Q27 – Thinks neighbours/friends would not like bouillon | 0.756 | Q25 – Positive views about study bouillon | 0.553 |
|  |  | Q28 – Wants household to use bouillon in future | 0.574 |
|  |  | Q29 – Would buy bouillon if sold in future | 0.692 |
| **Note**: All items exceeded the acceptable threshold for retention (standardised factor loading ≥ 0.40). Q7 (“Study bouillon can be used multiple times daily”) was excluded from the Acceptance construct during CFA due to a low standardised factor loading (0.35), resulting in a final 10-item construct. | | | |
